# Correction: Validation of a Polish version of the National Institutes of Health Stroke Scale: Do moderate psychometric properties affect its clinical utility?

**DOI:** 10.1371/journal.pone.0270016

**Published:** 2022-06-09

**Authors:** Adam Wiśniewski, Karolina Filipska, Marlena Puchowska, Katarzyna Piec, Filip Jaskólski, Robert Ślusarz

There are errors in the author affiliations. The correct affiliations are as follows:

Adam Wiśniewski^1*^, Karolina Filipska^2^, Marlena Puchowska^1^, Katarzyna Piec^1^, Filip Jaskólski^1^, Robert Ślusarz^2^

**1** Department of Neurology, Collegium Medicum in Bydgoszcz, Nicolaus Copernicus University in Toruń, Bydgoszcz, Poland,*Laboratory for Experimental Biotechnology*, *Laboratory for Experimental Biotechnology*, **2** *Laboratory for Experimental Biotechnology*, *Laboratory for Experimental Biotechnology*,Department of Neurological and Neurosurgical Nursing, Collegium Medicum in Bydgoszcz, Nicolaus Copernicus University in Toruń, Bydgoszcz, Poland.
