# Supplementary material for: Validation of a Polish version of the National Institutes of Health Stroke Scale: Do moderate psychometric properties affect its clinical utility?
Source: PLoS One. 2021 Apr 2;16(4):e0249211. doi: 10.1371/journal.pone.0249211 (PMC8018641; doi:10.1371/journal.pone.0249211)
Supplement: S1 Table — (PDF) [file pone.0249211.s001.pdf]

ITEM	SCORE
Poziom przytomności<br>(Level of consciousness)	0 – przytomny<br>(Alert)<br>1 - podsypiający, wybudza się przy niewielkiej stymulacji<br>(Not alert, but arousable with minimal stimulation)<br>2 - półśpiączka, wymaga wielokrotnej stymulacji<br>(Not alert, requires repeated stimulation to attend)<br>3 – śpiączka, brak reakcji na bodźce, wiotki, brak odruchów<br>(Coma)
Odpowiedź na pytanie<br>(level of consciousness questions)	0- obie odpowiedzi prawidłowe dotyczące miesiąca i wieku<br>(Answers both correctly)

	1- jedna odpowiedź prawidłowa<br>(Answers one correctly)<br>2- obie odpowiedzi nieprawidłowe<br>(Both incorrect)
Spełnianie poleceń<br>(level of connsiousness commands)	0 - oba polecenia wykonane prawidłowo<br>(Obeys both correctly)<br>1 - jedno polecenie wykonane prawidłowo<br>(Obeys one correctly)<br>2 - nie spełnia żadnego polecenia<br>(Both incorrect)
Ruchomość gałek ocznych<br>(Best gaze)	0 – prawidłowa, pełna ruchomość,<br>(Normal)<br>1 - częściowe porażenie<br>(Partial gaze palsy)<br>2 - przymusowe ustawienie gałek ocznych<br>lub całkowite porażenie<br>(Forced gaze palsy)
Pole widzenia<br>(Visual field testing)	0 - bez zaburzeń<br>(No visual field loss)

	1 - częściowe niedowidzenie połowicze<br>(Partial hemianopia)<br>2 - całkowite niedowidzenie połowicze<br>(Complete hemianopia)<br>3 - obustronne niedowidzenie połowicze<br>(Bilateral hemianopia)
Czynność nerwu twarzowego<br>(Facial palsy)	0 – prawidłowa ruchomość i symetria twarzy,<br>(Normal symmetrical movement)<br>1 - nieznaczny niedowład (spłycony fałd nosowo-wargowy, asymetryczny uśmiech)<br>(Minor paralysis (flattened nasolabial fold, asymmetry on smiling))<br>2 - częściowe porażenie (całkowite lub prawie całkowite porażenie dolnej części twarzy)<br>(Partial paralysis (total or near total paralysis of lower face))<br>3 - całkowite porażenie jedno- lub obustronne (dolnej i górnej części twarzy)<br>(Complete paralysis of one or both sides (abscence of facial movement in the upper and lower face))

<table>
<tr><td></td><td></td></tr>
<tr><td>Siła mięśniowa kończyn górnych
(Motor function arm)</td><td>0 - nie opada, chory utrzymuje kończynę pod kątem 90° (lub 45°) przez 10 s
(Normal (extends arm 90 or 45 degrees for 10 sec without drift))
1 - opada, chory utrzymuje kończynę pod kątem 90° (lub 45°) krócej niż 10 s
(Drift)
2- częściowy ruch przeciwko sile ciężkości
(Some effort against gravity)
3 - brak ruchu przeciwko sile ciężkości, kończyna opada
(No effort against gravity)
4 – całkowity brak ruchu
(No movement)
9 - amputacja, blok w stawie
(Joint fused/limb amputated)</td></tr>
<tr><td>Siła mięśniowa kończyn dolnych
(Motor function leg)</td><td>0 - pacjent utrzymuje kończynę pod kątem 30° przez 5 s
(Normal (holds leg in 30 degrees position for 5 sec without drift))</td></tr>
</table>

	1 – kończyna opada powoli przed upływem 5 s<br>(Drift)<br>2 – kończyna szybko opada przed upływem 5 s, ale zachowany jest opór przeciw sile ciężkości<br>(Some effort against gravity)<br>3 – kończyna opada natychmiast, ślad ruchu<br>(No effort against gravity)<br>4 - brak ruchu<br>(No movement)<br>9 - amputacja lub blok w stawie<br>(Joint fused/limb amputated)
Niezborność<br>(Limb ataxia)	0 - nieobecna w żadnej z kończyn<br>(No ataxia)<br>1 - obecna w jednej kończynie<br>(Present in one limb)<br>2 - obecna w obu kończynach<br>(Present in two limbs)<br>9 - amputacja lub blok w stawie<br>(Joint fused/limb amputated)

Czucie<br>(Sensory)	0 – prawidłowe<br>(Normal)<br>1 - łagodna lub umiarkowana utrata czucia<br>(Mild to moderate decrease in sensation)<br>2 - ciężka lub całkowita utrata czucia<br>(Severe to total sensory loss)
Ocena afazji<br>(Best language)	0 - brak afazji, mowa płynna, prawidłowa<br>(No aphasia)<br>1 - afazja łagodna do umiarkowanej<br>(Mild to moderate aphasia)<br>2 - afazja dużego stopnia<br>(Severe aphasia)<br>3 - całkowita afazja lub chory niemy<br>(Mute)
Ocena dyzartrii<br>(Dysarthria)	0 – brak cech dyzartrii, mowa wyraźna<br>(Normal articulation)<br>1 – dyzartria łagodna do umiarkowanej<br>(Mild to moderate slurring of words)

	2 – dyzartria ciężka, anartria<br>(Near unintelligible or unable to speak)<br>9 - chory zaintubowany lub inna bariera fizyczna<br>(Intubated or other physical barrier)
Reakcje na bodźce zewnętrzne jednoczesne<br>(Extinction and Inattention)	0 – prawidłowa reakcja na bodźce zewnętrzne,<br>(Normal)<br>1- połowiczy brak rozróżnienia działania bodźca jednego<br>rodzaju (czucia, słuchu, wzroku)<br>(Inattention or extinction to bilateral simultaneous stimulation in one of<br>the sensory modalities)<br>2 - połowiczy brak rozróżnienia więcej niż jednego typu bodźca<br>(Hemi-inattention, severe or to more than one modality)
