# Supplementary material for: Validation of a Polish version of the National Institutes of Health Stroke Scale: Do moderate psychometric properties affect its clinical utility?
Source: PLoS One. 2021 Apr 2;16(4):e0249211. doi: 10.1371/journal.pone.0249211 (PMC8018641; doi:10.1371/journal.pone.0249211)
Supplement: Attachment — Submitted filename: response to Reviewers.docx [file pone.0249211.s002.docx]

Response to Reviewers comments

**Academic Editor:**

The manuscript seems interesting, but I encourage the authors to seek for English Editing Services to correct grammatical errors through out the manuscript. Also, the quality of the images is low, and make them difficult to evaluate, please refer to PLOS ONE guidelines for images quality.

Response:

Dear Editor,

Thank You for this comment. According to the Editor recommendation our manuscript underwent the extensive language, style, grammar and typographical corrections made by Editage- Professional English Editing Service. We have also improved the quality and resolution of the Figures and checked them with PACE tool to ensure that they meet PLOS requirements. We also provided the changes suggested by the Reviewer, regarding stroke assessment at admission.

**Reviewer:**

The paper is interesting, well written and valuable. Results sound not too optimistic not too pessimistic, so PL-NIHSS may be used until new version of the scale will be prepared. It is first time done for Polish version. I have one important comment.
Authors are at day of admission, beside routine patients’ characteristic and PL- NIHSS are also doing mRS, Barthel and GCS. Writing in page 4, in methods. „which are most widely accepted tools for this purpose” not giving references. GCS may be done, but mRS and Barthel cannot be performed in most cases and is unreliable. At admission we ask patient or family and notice – pre stroke functional status.
I propose to delate these scales from admission characteristic, GCS can stay (not necessary), as well from results and figures. Is Ok to do mRS and Barthel after 90 days, but authors do not mention it in the methods, but we see in results.

Response:

Thank You for positive opinion regarding our paper.

As suggested by the Reviewer we have removed Barthel Index and modified Rankin Scale from admission characteristics, both in the Methodology Section, as well as in the Results Section and Figures. At this moment there are only PL-NIHSS and Glasgow Coma Scale as stroke assessment at admission. The stroke evaluation after 90 days is described in Methodology Section- Lines- 84-87.

I would like to thank You for the careful review of our study and the constructive comments that have been used to organize all issues and improve the work.

Początek formularza
